# A multiparametric approach to improve the prediction of response to immunotherapy in patients with metastatic NSCLC

**DOI:** 10.1007/s00262-020-02810-6

**Published:** 2020-12-14

**Authors:** Marzia Del Re, Federico Cucchiara, Eleonora Rofi, Lorenzo Fontanelli, Iacopo Petrini, Nicole Gri, Giulia Pasquini, Mimma Rizzo, Michela Gabelloni, Lorenzo Belluomini, Stefania Crucitta, Raffaele Ciampi, Antonio Frassoldati, Emanuele Neri, Camillo Porta, Romano Danesi

**Affiliations:** 1 Unit of Clinical Pharmacology and Pharmacogenetics, Department of Clinical and Experimental Medicine, University of Pisa, Pisa, Italy; 2 General Pathology, Department of Translational Research and New Technologies in Medicine, University of Pisa, Pisa, Italy; 3 Division of Translational Oncology, IRCCS Istituti Clinici Scientifici Maugeri, Pavia, Italy; 4 Diagnostic and Interventional Radiology, Department of Translational Research and New Technologies in Medicine, University of Pisa, Pisa, Italy; 5 Unit of Clinical Oncology, Specialist Medical Department, S. Anna University Hospital, Ferrara, Italy; 6 Endocrinology Unit, Department of Clinical and Experimental Medicine, University of Pisa, Pisa, Italy; 7 Department of Internal Medicine and Therapeutics, University of Pavia, Pavia, Italy; 8 Present Address: Unit of Medical Oncology, Department of Biomedical Sciences and Human Oncology, University of Bari ‘A. Moro’, Bari, Italy

**Keywords:** NSCLC, Liquid biopsy, Biomarkers, Radiomics, Immunotherapy

## Abstract

**Background:**

It is still unclear how to combine biomarkers to identify patients who will truly benefit from anti-PD-1 agents in NSCLC. This study investigates exosomal mRNA expression of PD-L1 and IFN-γ, PD-L1 polymorphisms, tumor mutational load (TML) in circulating cell-free DNA (cfDNA) and radiomic features as possible predictive markers of response to nivolumab and pembrolizumab in metastatic NSCLC patients.

**Methods:**

Patients were enrolled and blood (12 ml) was collected at baseline before receiving anti-PD-1 therapy. Exosome-derived mRNA and cfDNA were extracted to analyse PD-L1 and IFN-γ expression and tumor mutational load (TML) by digital droplet PCR (ddPCR) and next-generation sequencing (NGS), respectively. The PD-L1 single nucleotide polymorphisms (SNPs) c.-14-368 T > C and c.*395G > C, were analysed on genomic DNA by Real-Time PCR. A radiomic analysis was performed on the QUIBIM Precision^®^ V3.0 platform.

**Results:**

Thirty-eight patients were enrolled. High baseline IFN-γ was independently associated with shorter median PFS (5.6 months vs. not reached *p* = 0.0057), and levels of PD-L1 showed an increase at 3 months vs. baseline in patients who progressed (*p* = 0.01). PD-L1 baseline levels showed significant direct and inverse relationships with radiomic features. Radiomic features also inversely correlated with PD-L1 expression in tumor tissue. In subjects receiving nivolumab, median PFS was shorter in carriers of c.*395GG vs. c.*395GC/CC genotype (2.3 months vs. not reached, *p* = 0.041). Lastly, responders had higher non-synonymous mutations and more links between co-occurring genetic somatic mutations and ARID1A alterations as well.

**Conclusions:**

A combined multiparametric approach may provide a better understanding of the molecular determinants of response to immunotherapy.

**Supplementary Information:**

The online version contains supplementary material available at 10.1007/s00262-020-02810-6.

## Background

Anti-PD-1 therapies significantly improved the prognosis in a subset of patients affected by NSCLC. Despite several predictive biomarkers have been proposed, it is still unclear how to manage them in an integrated model to improve prediction power. PD-L1 assessment in tumor tissue has been widely used to identify patients who will benefit from immune-checkpoint inhibition; however, intratumor heterogeneity may cause false negative results [1] and patients with low PD-L1 expression may also take advantage from pembrolizumab [2]. Moreover, tumor phenotype, including PD-L1 expression, may vary throughout time in response to alterations in the tumor microenvironment and following the clonal selection induced by treatments [3–6]. For these reasons, other markers, such us PD-1/PD-L1 polymorphisms, tumor immune infiltrate, mutational load and microsatellite instability have been considered [7–9]. Moreover, several other mediators of the immune response have been investigated, such as IFN-γ, which has a pleiotropic activity on the immune system and can alter the expression of PD-L1 [10], albeit its role in tumor progression and immunotherapy response is still controversial [11]. To overcome spatial and temporal tumor heterogeneity, cell free tumor DNA and exosomes, due to their involvement in immune signalling, reprogramming of surrounding cells and immune escape, are gaining attention [12–15]. In addition to this, the concept that biomedical images contain information reflecting tumor molecular aberrations is nowadays raising, and radiomic analysis is being used to identify predictive biomarkers of response to treatments [16, 17]. For these reasons, the present study was aimed at integrating the evaluation of: (1) PD-L1 and IFN-γ mRNA expression in plasma-derived exosomes; (2) selected PD-L1 gene variants (i.e. c.-14-368 T > G and c.*395G > C); (3) tumor mutational load (TML) on cfDNA, and (4) radiomic analysis to identify predictive biomarkers of response to anti-PD-1 therapy.

## Methods

### Patients

Patients affected by locally advanced or metastatic NSCLC given nivolumab or pembrolizumab as per approved schedule were enrolled in the present study. Blood samples were drawn from each patient for the analysis of (1) exosomal mRNA levels of PD-L1 and IFN-γ at time 0 (baseline) and after 3 months of treatment, (2) selected germinal PD-L1 polymorphisms and (3) tumor mutational load (TML) in circulating free DNA (cfDNA). PD-L1 immuno-histochemistry assessment in tumor tissue was collected in selected cases, accordingly with laboratories procedures. Complete (CR) and partial response (PR), disease stabilisation (SD) and disease progression (PD) were defined following RECIST (v. 1.1) criteria. CT scans were collected at baseline for radiomic analysis (see below). A written consent form was obtained from all patients; the study was approved by the local Ethics Committee and performed in accordance with the provisions established by the Helsinki Declaration.

### Exosomes isolation and measurement of PD-L1 and IFN-γ mRNA

A blood sample of 12 ml was collected in EDTA tubes and centrifuged for 10 min at 1900 g within 2 h. Exosomes were isolated from plasma and RNA was extracted, as previously described [18]. Expression of PD-L1 and IFN-γ was assed via ddPCR (Bio-Rad, Hercules, CA) with respect to human β-actin (ACTB) as internal control, as previously described [4]. The values reported are expressed as fractional abundance (FA, %), that is the proportion of the number of copies/ml of the investigated protein-coding exosomal mRNAs in the total of the commonly detected exosomal mRNAs, including also  β-actin (ACTB) as housekeeping gene, calculated by the QuantaSoft™ software (Bio-Rad, Hercules, CA).

### Analysis of PD-L1 germline polymorphisms

Germline DNA was extracted from 200 μl of peripheral blood (EZ1 Extractor; Qiagen, Valencia, CA) for the analysis of the PD-L1 single nucleotide polymorphisms (SNPs) c.*395G > C and c.-14-368 T > C, selected on the basis of a previous publication [9, 19], by a real-time PCR using the TaqMan^®^ SNP Genotyping Assay (ThermoFisher, Carlsbad, CA).

### TML analysis on cfDNA

A blood sample of 6 ml was collected in EDTA tubes and centrifuged for 10 min at 1900 g within 2 h. cfDNA was extracted from 3 ml of plasma and DNA was eluted in 50 μl of buffer, as previously published [20]. TML analysis was performed on the Ion S5 XL System NGS platform, using the Oncomine Tumor Mutation Load Assay (ThermoFisher, Carlsbad, CA). The filtered variants were examined using the Integrative 201 Genomic Viewer IGV tool to check their quality level and confirm the presence of the variant of interest. To explore the relationship between the quality of genetic profiles and TML, all genes and relative calling mutations were evaluated according to their involvement in NSCLC pathogenesis, immune system, cell cycle, and immunotherapy response. For each SNV (single-nucleotide variant) occurring in the exonic region, an estimation of its putative damaging effect on the resulting protein was scored using Grantham, SIFT and PolyPhen criteria [21]. Finally, a computational approach was attempted [22, 23]. Starting from Milgram's basic small-world concept [22] a custom-made MATLAB^®^ script (The Math Works Inc., Natick, MA) was endeavored to investigate possible relationships between genes included in the Oncomine panel. For each gene the total number of different mutations was considered, weighed by the number of mutations with Polyp hen ≥ 0.85 (deleterious power) and a Watts–Strogatz graph [23] was plotted.

### Radiomic analysis

Patients who undergone to CT imaging for lung cancer staging and met homogeneity criteria for image acquisition parameters [24], were retrospectively enrolled in a radiomics analysis evaluating 25 radiomics features from the entire primary tumor lesion at baseline (Supplementary Table S1). Scan protocol homogeneity criteria included 120 kV tube voltage, a field of view between 36 and 40 cm, 1.5–2 mm slice thickness and a standard/soft tissue convolution kernel. Only non-contrast CT images were used for radiomics analysis. CT scan were acquired at baseline of immunotherapy, either first-line pembrolizumab or nivolumab as subsequent-line. The primary tumor site was manually contoured slice by slice on axial CT images using a lung window setting (width, 1500 HU; level, − 600 HU) by a radiologist experienced in lung cancer imaging, and then independently validated by another radiologist assessor. Radiomic analysis was then performed on the volume of interest (VOI) via the QUIBIM Precision^®^ V3.0 platform (QUIBIM SL, Valencia, Spain) [25]. Lastly, a statistical method was used to avoid a redundancy of information [26] and to select only the most distinctive radiomic features (Supplementary Table S2).

### Statistical analysis

Categorical variables were described by absolute and relative frequencies while quantitative factors by median and range. To compare quantitative with categorical variables the Mann–Whitney test was performed, while the Wilcoxon’s test was used to assess paired data. The median cut-off value for analysis of PD-L1 and IFN-γ was calculated by the Receiver Operating Characteristic (ROC) curve analysis to differentiate patients with response and no response to ICIs. Since an overall survival (OS) advantage was difficult to detect due to the small sample size and the short follow-up, progression free survival (PFS), overall response rate (ORR) and clinical benefit rate (CBR) were investigated [27, 28]. Moreover, CBR and PFS are generally based on objective and quantitative assessments, including the measurement of stable disease, and are not affected by crossover or subsequent therapies. PFS was defined as the time from treatment start to PD or death. ORR was defined as the proportion of patients achieving CR and PR. CBR was defined as the proportion of patients achieving CR, PR or SD for at least 24 weeks. Log-rank test was used to evaluate differences between curves and hazard ratio was calculated using Cox model to compare cumulative risks. Pearson’s correlation coefficient was used to assess the correlation among all radiomic features, and between radiomic features and available molecular data. Lastly, a radiomic signature was calculated based on the sum of the features correlating with tumor molecular data, weighted by their corresponding maximum-likelihood fitted coefficients for the least absolute shrinkage and selection operator (LASSO) regression model. A 11-fold cross validation was performed for this purpose. Logistic regression model test (Cox-Snell’s *R*^2^) and receiver operating characteristic (ROC) curve analysis was computed to estimate diagnostic performance of such signatures, alone and combined with the other biomarkers analysed. Differences were considered significant at *p* < 0.05. All statistical analyses were performed with SPSS version 24 (SPSS Inc. SPSS^®^ Chicago, IL, USA) or MatLab^®^ software (version R2019a; MatLab, The Math Works Inc., Natick, MA).

## Results

### Patients’ characteristics

A total of 38 NSCLC patients were enrolled in the study (Table 1). Twenty-five patients received nivolumab as second (68%) or higher line of treatment (32%), while 13 patients received pembrolizumab as first line treatment. At the first radiological assessment 13 patients (34.2%) obtained a PR, 8 (31.6%) SD and 17 (44.8%) PD. ORR was 34%, slightly different amongst the two cohorts (53% vs. 24% for pembrolizumab and nivolumab, respectively), CBR was 66% (85% vs. 56% for pembrolizumab and nivolumab, respectively).

**Table 1:** Clinical characteristics of patients

	Overall population (*n* = 38)	Nivolumab (*n* = 25)	Pembrolizumab (*n* = 13)
Age, mean (range)	68 (44–85)	72 (52–85)	65.5 (44–74)
Sex, *n* (%)			
Male	22 (57.9)	13 (52)	9 (69.2)
Female	16 (42.1)	12 (48)	4 (30.8)
Smoking habits, *n* (%)			
Yes/former	34 (89.5)	22 (88)	12 (92.3)
Never	4 (10.5)	3 (12)	1 (7.7)
ECOG PS, *n* (%)			
0–1	38 (100)	25 (100)	13 (100)
≥ 2	0	0	0
Stage, *n* (%)			
III	0	0	0
IV	38 (100)	25 (100)	13 (100)
Sites of disease, mean (range)	2 (1–5)	2.5 (1–5)	2 (1–5)
Site of disease			
Bone, *n* (%)	9 (23.7)	6 (24)	3 (23.1)
Visceral, *n* (%)	6 (15.8)	5 (20)	1 (7.7)
Central nervous system, *n* (%)	2 (5.3)	2 (8)	0
Line of therapy, *n* (%)			
I	8 (21.1)	0	8 (61.5)
≥ II	30 (78.9)	25 (100)	5 (38.5)

*ECOG PS* eastern cooperative oncology group performance status, *n* number

### Association between mRNA expression of PD-L1 and IFN-γ and clinical outcome

**Fig. 1:**
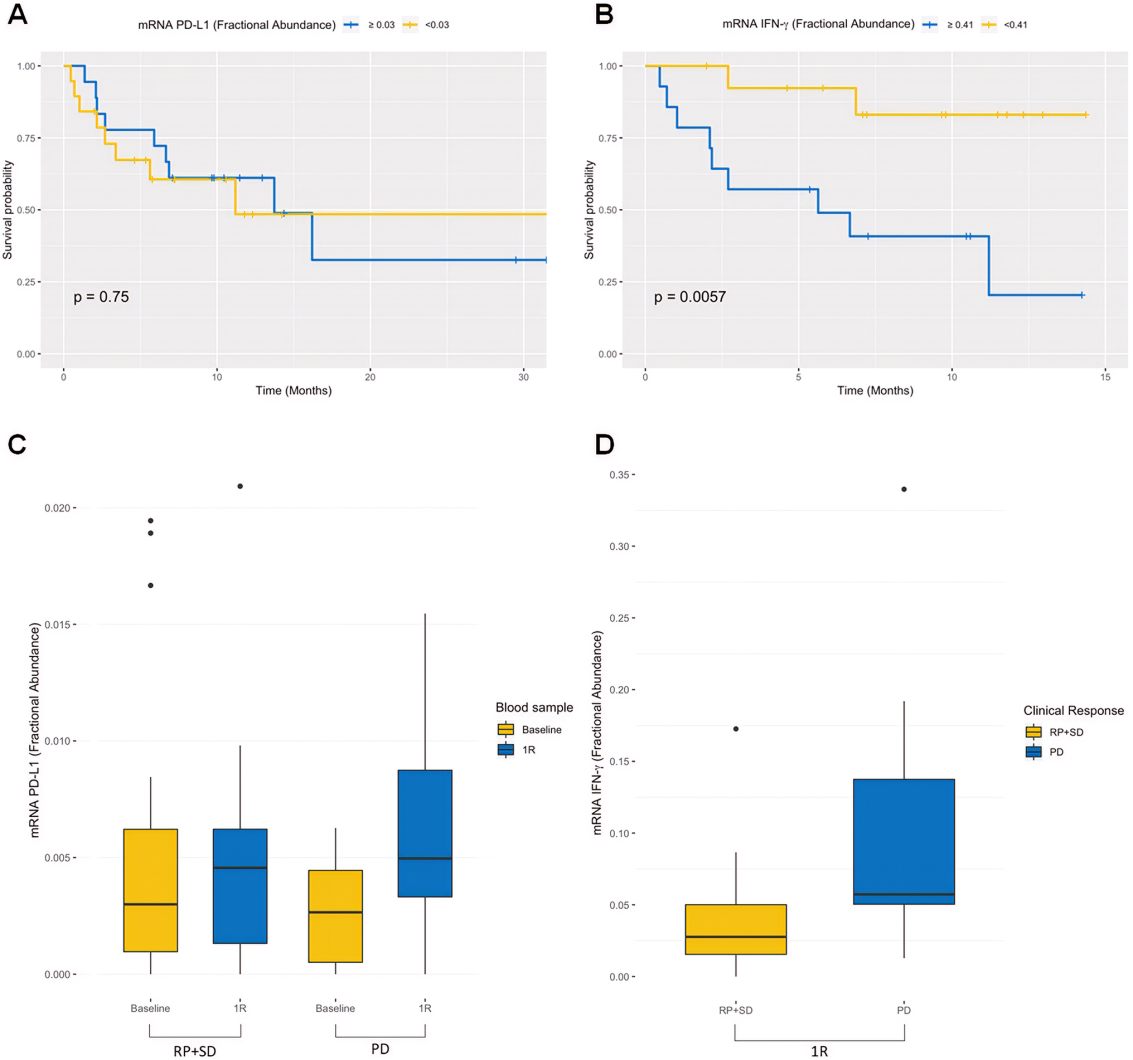
Kaplan–Meier PFS of 38 NSCLC patients stratified on the basis of fractional abundance of PD-L1 (**a**) and IFN-γ (**b**). Comparison of fractional abundance of PD-L1 (**c**) and IFN-γ (**d**) among patients with PR, SD and PD, at baseline and after first evaluation (1R)

Median FA at baseline was 0.3% for PD-L1 and 4.1% for IFN-γ (overall population, 38 patients). PFS was 11 vs. 16.2 months in patients with PD-L1 FA of < 0.3% vs. ≥ 0.3%, respectively (*p* = 0.75; Fig. 1a). PFS was 5.6 months vs. not reached in patients with baseline FA of IFN-γ of ≥ 4.1% vs. < 4.1%, respectively (*p* = 0.0057; Fig. 1b). Comparing PD-L1 at baseline vs. 3 months of treatment, the FA was significantly increased in patients with PD vs. PR + SD (*p* = 0.01; Fig. 1c). Of note, considering IFN-γ baseline levels, the FA was significantly higher in patients who progressed within 3 months compared to patients with PR + SD (*p* = 0.04; Fig. 1d).

### Association between PD-L1 polymorphism and clinical outcome

**Fig. 2:**
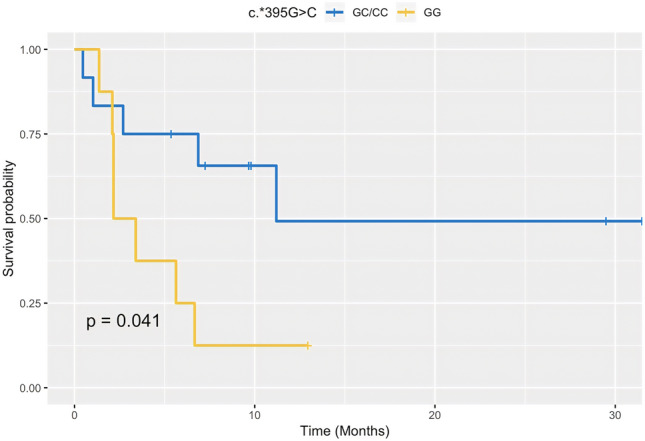
Kaplan–Meier curve of PFS in 20 NSCLC patients treated with nivolumab according to the PD-L1 c.*395G > C polymorphism

c.-14-368 T > G and c.*395G > C PD-L1 polymorphisms were obtained in 32 out of 38 patients (20 patients treated with nivolumab and 12 with pembrolizumab). The absolute and relative frequencies are reported in Table 2. In the cohort treated with nivolumab, median PFS was significantly shorter in patients with c.*395GG vs. those carrying the c.*395GC/CC genotype (2.3 months vs. not reached, *p* = 0.041; Fig. 2). No significant association between c.*395G > C genotypes and PFS was observed in cohort of patients given pembrolizumab and in the overall population. The c.-14-368 T > G genotype was not correlated with PFS neither in the overall population nor in the cohorts treated with nivolumab or pembrolizumab alone.

**Table 2:** PD-L1 genotypes and clinical response at 3 months evaluation

Clinical response	c.*395G > C (*n* = 32)			c.-14-368 T > G (*n* = 32)		
	GG	GC	CC	TT	TG	GG
PR, *n* (%)	6 (18.8)	6 (18.8)	1 (3.2)	5 (15.6)	6 (18.8)	2 (6.2)
SD, *n* (%)	3 (9.4)	4 (12.5)	1 (3.2)	5 (15.6)	3 (9.4)	0
PD, *n* (%)	8 (25)	3 (9.4)	0	4 (12.5)	5 (15.6)	2 (6.2)

*n* number, *T* time, *PR* partial response, *SD* stable disease, *PD* progression disease

### TML and mutated gene network

TML analysis on cfDNA was available  only in cfDNA samples showing a sufficient concentration of 150–300 bp DNA. PD was associated with lower mutation load compared to patients who achieved a PR (Supplementary Table S3). Furthermore, for all the exonic SNVs of each patient, the relative median values of Grantham, PolyPhen and SIFT scores were obtained. Subjects who underwent PR had higher Grantham, PolyPhen and SIFT mean values than the patient in PD (67.17 ± 6.53 vs. 50, 0.01 vs. 0, 0.90 ± 0.04 vs. 0.80, respectively).

**Fig. 3:**
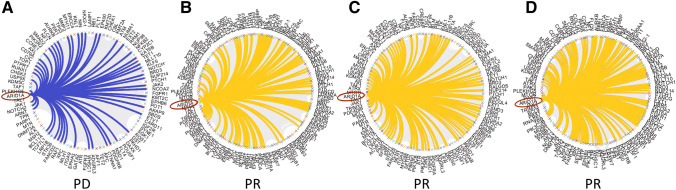
Watts–Strogatz graph showing the connections between ARID1A and other genes included in the TML NGS panel according to clinical response

Likewise, in the underlying architecture among mutated genes defining the TML, fewer connections were evident between mutated AT-Rich Interaction Domain 1A (ARID1A), a gene involved in transcriptional regulation and DNA damage response, and the other genes in the patient who had PD with respect to those who achieved PR (Fig. 3).

### Radiomic results

Radiomic analysis was performed in 11 subjects (Supplementary Fig. S1). FA of PD-L1 at baseline directly correlated with contrast (*p* = 0.96, *p* = 0.003), dissimilarity (*p* = 0.92, *p* = 0.008) and sum variance (*p* = 0.91, *p* = 0.009), and inversely with entropy (*p* = − 0.95, *p* = 0.004). Inverse correlations were also found between PD-L1 expression in tumor tissue with autocorrelation (*p* = − 0.83, *p* = 0.04) and sum average (*p* = − 0.82, *p* = 0.05).

Notably, tissue expression of PD-L1 was variable; in 45% of subjects the PD-L1 tumor proportional score (TPS) was < 1%, in 27.5% was in the 1–49% range and in 27.5% was ≥ 50%. Two of them had levels ≥ 75%.

**Fig. 4:**
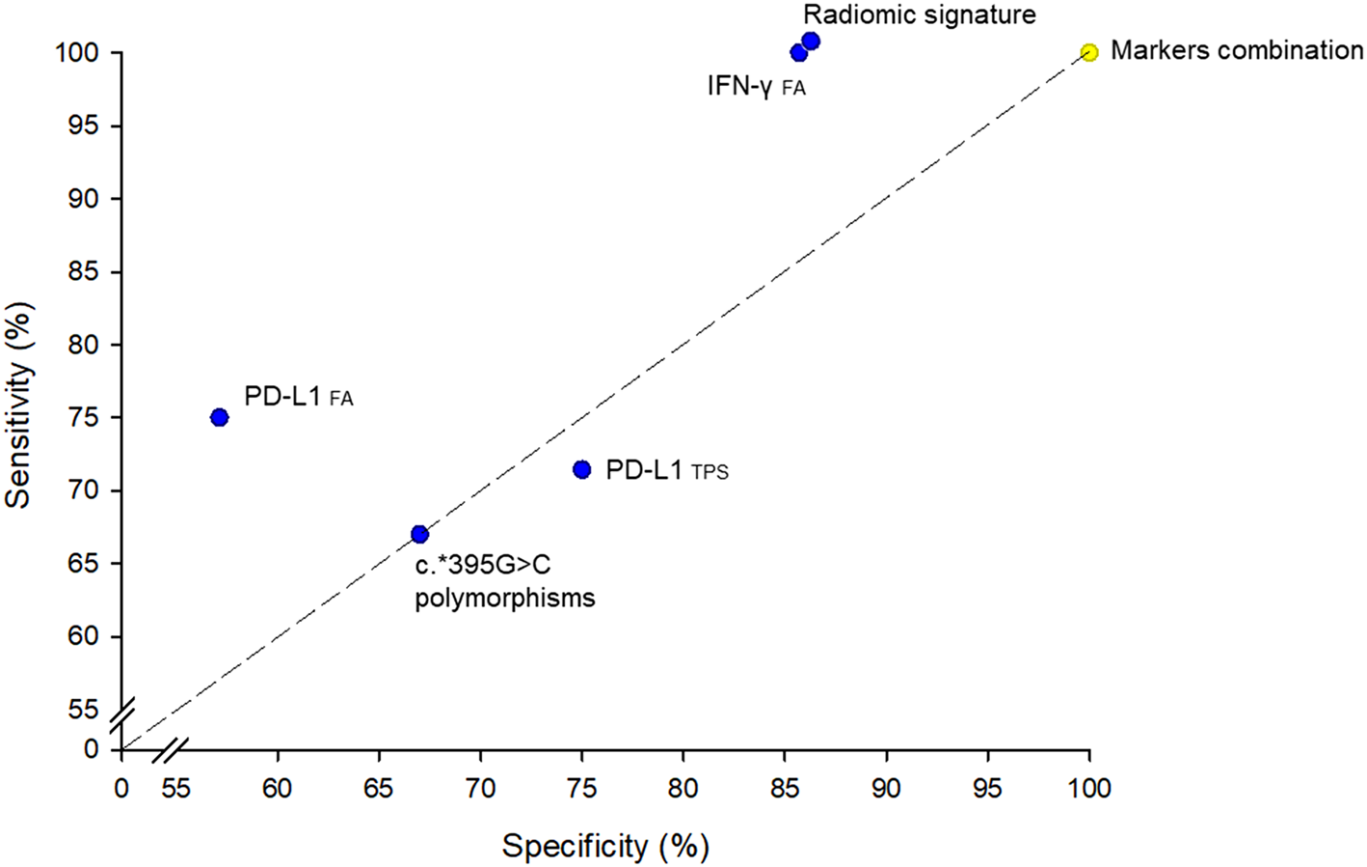
Specificity and sensitivity of radiomic signature, PD-L1 and IFN-γ FA and c.*395G > C polymorphism

No correlations were found between radiomic features and IFN-γ FA. The radiomic signature comprising the abovementioned 6 features evidenced good capability with acceptable representativeness for predicting patients in PD vs. those who underwent PR or SD (Cox-Snell’s *R*^2^ = 0.63, *p* < 0.001). The optimal cut-point estimated from the ROC curve showed 85.71% sensitivity and 100% specificity, with the area under the curve of 0.96. Combining the signature with the other biomarkers, representativeness improved (Cox-Snell’s *R*^2^ = 0.72, *p* = 0.02) and ROC curve showed sensitivity and specificity of 100%. A multiparametric analysis gave a better diagnostic performance than single parameters: with regard to clinical response, the sensitivity and specificity were, respectively, 75% vs. 57.14% for FA of PD-L1, 100% vs. 85.71% for FA of IFN-γ and 71.43% vs. 75% for PD-L1 TPS cutoff of 50% (Fig. 4).

## Discussion

The availability of predictive biomarkers for immunotherapy response is still an important need in many solid tumors. Many efforts have been made to identify a reliable marker; however, except for the PD-L1 expression of patients candidate to first line treatment with pembrolizumab, no robust biomarkers have been identified. Even among NSCLC patients with PD-L1 expression > 50% treated with first-line pembrolizumab, clinical outcome resulted significantly improved in patients with a PD-L1 expression > 90% [29]. While drivers such as EGFR, BRAF, ALK are HER2 dictate the choice of target-specific therapy, the same cannot be attributed to PD-L1 because it is dynamic, inducible, and disease-dependent. Therefore, it is reasonable that more than one biomarker is needed to select patients who will benefit or not from immunotherapy. Based on this hypothesis, in this study we considered exosomal mRNA expression of PD-L1 and IFN-γ, together with cfDNA-derived TML and radiomic features as a possible pool of predictive markers of response to the anti-PD-1 agents nivolumab and pembrolizumab in NSCLC.

The baseline expression of IFN-γ was significantly higher in patients who progressed. Indeed, although IFN-γ has long been implicated as a central orchestrator of antitumor immune responses [30], mounting evidence [31–33] suggests that it may also have a pivotal role in immune evasion. IFN-γ upregulates PD-L1 expression in cancer, stromal and myeloid cells, impairing immune response [34]. Both CD8 + T-cells and IFN-γ are critical for antitumor immunity [35], but a prolonged IFN-γ signaling in tumors, coordinates resistance to immune checkpoint inhibitors through a multigenic resistance program [36] independent of PD-L1 expression. Different studies evaluated the role of inflammatory cytokines as predictive biomarkers, although results are controversial [37]. The emerging picture of the immune landscape of NSCLC has provided evidence for an extremely high degree of complexity and heterogeneity [38]. Considering such heterogeneity, looking at only few markers (such as PD-L1) could be not robust enough. For these reasons, the development of a multiparametric approach is an emerging challenge to select patients more likely to respond. The feasibility to assess TML on cfDNA was evaluated, and a radiomic analysis has been conducted in this study. It is well known that tumors with high TML and responsive to immunotherapy may exhibit specific non-synonymous genetic alterations [39, 40]. For example, defects in MMR genes lead to MSI and could cause an increase in TML [39, 41]. In this study, albeit in a small cohort, patients who achieved a PR had higher amount of non-synonymous exonic mutations than the patient who underwent PD. Moreover, Grantham, SIFT and PolyPhen scores were higher in patients with PR, indicating a possible biological condition consisting of numerous damaging mutations, compared with those found in the progressing patient. It is likely that an immunogenic phenotype may arise, leading to better responsiveness to immunotherapy. Of note, in the underlying architecture among mutated genes included in the NGS panel used to assess TML, patients who achieved a PR showed higher connections between mutated ARID1A gene and the others with respect to the one who underwent PD (Fig. 3). A pan-cancer analysis of ARID1A alterations [42] recently highlighted their important value as predictive biomarkers for immunotherapy. ARID1A alterations promote cancer iper-mutated phenotype [43, 44] and co-occurring specific genetic mutations in cancers with ARID1A alterations are detected [42]. Looking at differences in the number of ARID1A connections between PD and PR patients may not only suggests a possible novel marker to be considered in patients treated with immunotherapy, but also confirms the role of ARID1A in promoting immunogenicity. Currently, TML measurements are essentially performed using NGS in tissue biopsy and the cut-off can vary from 5 to 10% [45]. TML measurements from cfDNA is technically challenging, due to the low quantity and quality of cfDNA that can be extracted from plasma. Therefore, the identification of the cut-off of variant allele frequency is mandatory to ensure that TML would be a reliable estimation of the number of the mutations in the tumor, avoiding false negative/positive results.

The choice of using a targeted-NGS panel instead of whole-exome sequencing (WES), have been carefully evaluated, since the panel size and the kind of included variants for TML analysis remain a key question [46, 47], and a recent report described that in panels with genomic coverage < 0.5 Mb, the accuracy of TML determined by targeted NGS diminished [48]. Nevertheless, our approach was intended to meet the clinic-laboratory need of cost-efficiency. Several studies show that TML measured by WES is not currently feasible in routine clinical setting due to high costs, long turnaround time and limited availability of samples [49]. Interesting, previous works demonstrated the comparability of panel-based sequencing versus WES in NSCLC patients treated with immunotherapy: Ritzvi H. et al. [50], performing WES and targeted NGS for 49 patients, showed a significant correlation between the two methodologies (*R* = 0.86; *p* < 0.001); similarly, another study by Wang Z. et al. [51] demonstrated the same correlations (median *R*^2^ = 0.92; interquartile range = 0.91–0.93). Moreover, the researchers also confirmed that TML may be a potential biomarker to identify patients who will benefit from anti-PD-1/PD-L1 therapy.

The present study was also aimed at evaluating the impact of germline SNPs of PD-L1 (c.-14-368 T > G and c.*395G > C) on PFS as previously investigated [9]. In the present work, patients treated with nivolumab showed a shorter median PFS in the c.*395GG wild type carriers, compared with those carrying the mutant c.*395GC/CC genotype. The c.*395G > C polymorphism increases promoter activity and PD-L1 mRNA levels and is significantly associated with better survival. These findings suggest that PD-L1 polymorphism may be useful predictor of response to treatment [19, 52]. Moreover, our results showed that PD-L1 expression in exosomes is not a predictive biomarker of response at baseline; however, PD-L1 expression was found to increase in patients who progressed to treatment, confirming the results of a previous published study [4]. Given the complexity and multifactorial nature of the anticancer immune response and the mechanisms of tumor immune evasion [38], finding a reliable signature that allows distinguishing patients who will benefit from immunotherapy is still a mission. In this context, radiomics could be a useful tool, as it allows to highlight alterations of neoplastic texture [53, 54]. Radiomics has the potential to provide an individualized quantitative (and therefore objective) measurement of tissue reaction to treatment in terms of tumor response [17], which cannot be accurately derived by either human visual assessment or laboratory data, alone. However, the significance of the association between radiomics and the complex biological processes occurring within the tumor also remains challenging. Therefore, combined molecular and radiomic data could help to clarify the meaning of imaging-based features and increase the predictive significance of bench results. In our study Pearson’s correlation showed a direct relation between exosomal mRNA expression of PD-L1 and radiomic features such as “contrast”, “dissimilarity” and “sum variance”, while an inverse correlation was showed between PD-L1 expression and “entropy”. Moreover, an inverse correlation was also found for PD-L1 expression in tumor tissue with the “autocorrelation” and “sum average” features. Data from this preliminary study show that liquid biopsy can be completed by radiomic features, which could reflect tumor spatial heterogeneity, microenvironment and mutational profile [55]. Although the results emerge from the analysis of 11 patients only, and need to be confirmed in a larger population, they are promising and could be intended to underline the valuable contribution of texture analysis in a multi-parametric context. Our results are consistent with another study from the University of Oklahoma reporting that while radiomics (AUC = 0.78) and genomics (AUC = 0.78) models were capable of predicting survival, accuracy significantly improved (AUC = 0.84) when both data were combined [56].

In the present study a manual segmentation of the VOIs has been adopted, even if it is labour intensive and not always feasible for radiomic analysis, since it requires very large data-sets. Moreover, many authors consider manual segmentation by expert readers the ground truth despite high inter-reader variability [57–60]. However, it is not clear to what degree segmentation variability has an impact on radiomics features, even considering that a universal automatic segmentation algorithm has not been validated and established for all image applications, and some features may not show stability and reproducibility using different methods. Furthermore, automatic segmentation means “probabilistic” segmentation, and the ground truth for automatic boundering comes only for big datasets able to train the neural network [61]. Unfortunately, 11 patients were not sufficient to apply an automatic or semi-automatic approach, leading to more variable results than manual segmentation. The number of enrolled patients for radiomic analysis and the type of lesions to be contoured, however, allowed the use of a handcrafted system for identification and segmentation of primary lesions. Indeed, lung tumours present as homogenous, high-intensity lesions on a background of low-intensity lung parenchyma [62, 63] and, therefore, can be manually segmented with high reproducibility and accuracy.

The analysis of different potential biomarkers using sophisticated technologies, represent the major advantages of this study. However, limitations should be also acknowledged: first, some of the examined potential biomarkers have been explored in a limited number of patients, due to the suboptimal yield of genetic material; second, NSCLC patients were included from both the first- and second-line setting, and immune checkpoint inhibitor received was consequently different. Therefore, using anti-PD-1 blockade in different lines of treatment could have been a bias. Nonetheless, multiple studies have investigated the survival benefit of immunotherapy when administered in different lines for patients with NSCLC. As an example, Paz-Ares et al. [64] and Gandhi et al. [65] in 2018, as well as Borghaei et al. in 2019 [66], showed the efficacy of first-line ICIs in both PFS (HR: 0.56 95% CI 0.45–0.70; HR: 0.52 95% CI 0.43–0.64; HR: 0.53 95% CI 0.33–0.86, respectively) and OS (HR: 0.64 95% CI 0.49–0.85; HR: 0.49 95% CI 0.38–0, 64; HR: 0.56 95% CI 0.32–0.95, respectively), and similar results also emerged from OS and PFS analysis by Brahmer et al. [67] and Herbst et al. [68] investigating ICIs in the sub-sequent line setting (HR PFS: 0.62 95% CI 0.47–0.81 and HR OS: 0.59 95% CI 0.44–0.79; HR PFS: 0.79 95% CI 0.66–0.94 and HR OS: 0.71 95% CI 0.58–0.88, respectively). These results suggested that anti-PD-1 inhibitors provide longer PFS and OS both when used as first-line treatment and as a subsequent-line setting, with no substantial differences. Unfortunately, the sample size did not allow to come up with a signature that can be associated with a reliable ORR, and a prospective study with larger population is needed to confirm these preliminary data.

In conclusion, the study of correlations between radiomic features and tumor molecular data may offer a reliable picture of the pathophysiological processes underlying cancer progression, better than single parameters considered individually. The present results confirm the predictive role of a  combined approach using genomic and imaging-based data to capture both disease heterogeneity and dynamic changes induced by treatment, suggesting a novel approach in patient management.

If validated in larger and prospective studies, the immune-radiogenomic analysis may thus help in understanding of the molecular determinants of response to immunotherapy.

### Supplementary Information

Below is the link to the electronic supplementary material.

Supplementary file1 (PDF 154 KB)

Supplementary file2 (PDF 80 KB)

Supplementary file3 (PDF 89 KB)

Supplementary file4 (PNG 3657 KB) Supplementary Fig. S1. Heatmaps describing Spearman's correlation between radiomic features and molecular parameters. Bright yellow indicates direct correlation while blue the inverse one, with a gradient from yellow to blue indicating the Spearman's correlation coefficient. Abbreviation: FA, fractional abundance

## Data Availability

The dataset used in the current study is available as unpublished material, if requested. MDR, FC and RD had full access to all the data in the study and takes responsibility for the integrity of the data and the accuracy of the data analysis.

## Abbreviations

ACTB: Human β-actin
ARID1A: AT-Rich Interaction Domain 1A
bp: Base pair
c.*395G > C: 395 Nucleotides downstream of the stop codon guanine to cytosine substitution
c.-14-368 T > G: Intron nucleotides upstream of the start codon thymine to guanine substitution
CBR: Clinical benefit rate
cfDNA: Circulating cell-free DNA
CR: Complete response
ddPCR: Droplet digital polymerase chain reaction
EDTA: Ethylenediamine tetra-acetic acid
FA: Fractional abundance
HU: Hounsfield Unit
IFN-γ: Interferon gamma
LASSO: Least absolute shrinkage and selection operator
MMR: Mismatch repair
MSI: Microsatellite instability
NGS: Next-generation sequencing
NSCLC: Non-small cell lung cancer
ORR: Overall response rate
PD: Progression disease
PD-1: Programmed cell death protein-1
PD-L1: Programmed cell death protein–ligand 1
PFS: Progression free survival
PolyPhen: Polymorphism phenotyping
PR: Partial responses
RECIST: Response evaluation criteria in solid tumors
ROC: Receiver operating characteristic
SD: Stable disease
SIFT: Sorting intolerant from tolerant
SNPs: Single nucleotide polymorphisms
SNV: Single-nucleotide variant
TML: Tumor mutational load
TPS: Tumor proportional score
VOI: Volume of interest
