# Supplementary material for: A multiparametric approach to improve the prediction of response to immunotherapy in patients with metastatic NSCLC
Source: Cancer Immunol Immunother. 2020 Dec 14;70(6):1667–78. doi: 10.1007/s00262-020-02810-6 (PMC8139911; doi:10.1007/s00262-020-02810-6)
Supplement: Supplementary file 1 — Supplementary file1 (PDF 154 KB) [file 262_2020_2810_MOESM1_ESM.pdf]

Supplementary Table S1. A summary describing the radiomic features extracted from CT images

Feature type	Feature name	Description
**Shape**<br>*Describing the three-dimensional physical appearances of the tumor*	Volume	
**First-order statistics**<br>*Quantifying properties of the voxel intensity histogram*	GL Mean, STD	
	GL Median, p25, p75	
	GL Skewness	Measure of lopsidedness of the intensity distribution
	GL Kurtosis	Measure of the heaviness of the tail of the intensity distribution
**Texture**<br>*Quantifying the spatial relationships between voxel intensities.*	Autocorrelation	Measure of texture fineness and coarseness
	Cluster prominence	Measure of image asymmetry of the GLCM
	Cluster shade	Measure of the skewness of the GLCM
	Contrast	Measure of the local variations presented in the image
	Correlation	Measure of the linear dependency of image intensity of the neighboring voxels
	Difference entropy	Measure of the variability in neighboring intensity value differences
	Difference variance	Measure of heterogeneity
	Dissimilarity	
	Energy	Measure of homogeneity of an image
	Entropy	Measure of image texture randomness
	Homogeneity	
	Inverse difference	Measure of the local homogeneity of an image
	Maximum probability	The number of most occurred pair of neighboring intensity values
	Sum average	Average value of the GLCM
	Sum entropy	Measure of randomness of the GLCM
	Sum squares	Measure of the neighboring intensity level pairs about the mean GLCM intensity level
	Sum variance	High weight on the elements different from the GLCM average value

The Gray Level Co-occurrence Matrix (GLCM) considers the spatial relationship of pixels with specific intensity. Abbreviation: GL, Grey Level; STD, Standard Deviation; p25, 25th percentile; p75, 75th percentile; GLCM, Gray Level Co-occurrence Matrix.
