# Supplementary material for: A multiparametric approach to improve the prediction of response to immunotherapy in patients with metastatic NSCLC
Source: Cancer Immunol Immunother. 2020 Dec 14;70(6):1667–78. doi: 10.1007/s00262-020-02810-6 (PMC8139911; doi:10.1007/s00262-020-02810-6)
Supplement: Supplementary file 2 — Supplementary file2 (PDF 80 KB) [file 262_2020_2810_MOESM2_ESM.pdf]

Supplementary Table S2. Selected radiomic features

Feature type	Feature name
**Shape**	Volume
**First-order statistics**	GL Mean, STD
	GL Median
	GL Skewness
	GL Kurtosis
**Texture**	Autocorrelation
	Contrast
	Correlation
	Dissimilarity
	Entropy
	Homogeneity
	Maximum probability
	Sum average
	Sum entropy
	Sum variance

Abbreviation: GL, Grey Level; STD, Standard Deviation
