# Supplementary material for: A multiparametric approach to improve the prediction of response to immunotherapy in patients with metastatic NSCLC
Source: Cancer Immunol Immunother. 2020 Dec 14;70(6):1667–78. doi: 10.1007/s00262-020-02810-6 (PMC8139911; doi:10.1007/s00262-020-02810-6)
Supplement: Supplementary file 3 — Supplementary file3 (PDF 89 KB) [file 262_2020_2810_MOESM3_ESM.pdf]

**Supplementary Table S3. Tumor mutational load (TML)**

| | PD | PR |
|---|---|---|
| **Counts, n** | 1 | 3 |
| **Somatic, mut** | 456 | 639.67 ± 107.55 |
| **Synonymous mutation, mut** | 290 | 415.33 ± 79.48 |
| **Non-synonymous mutation, mut** | 166 | 224.33 ± 30.44 |
| **Total mutation, mut/Mb** | 278.39 | 391.62 ± 65.41 |

Data presented as mean ± STD.
Abbreviation: STD, standard deviation; n, number; mut, mutations; mut/Mb, mutations per megabase; PR, partial response; PD, progression disease
