# Supplementary figures and images for: A multiparametric approach to improve the prediction of response to immunotherapy in patients with metastatic NSCLC
Source: Cancer Immunol Immunother. 2020 Dec 14;70(6):1667–78. doi: 10.1007/s00262-020-02810-6 (PMC8139911; doi:10.1007/s00262-020-02810-6)

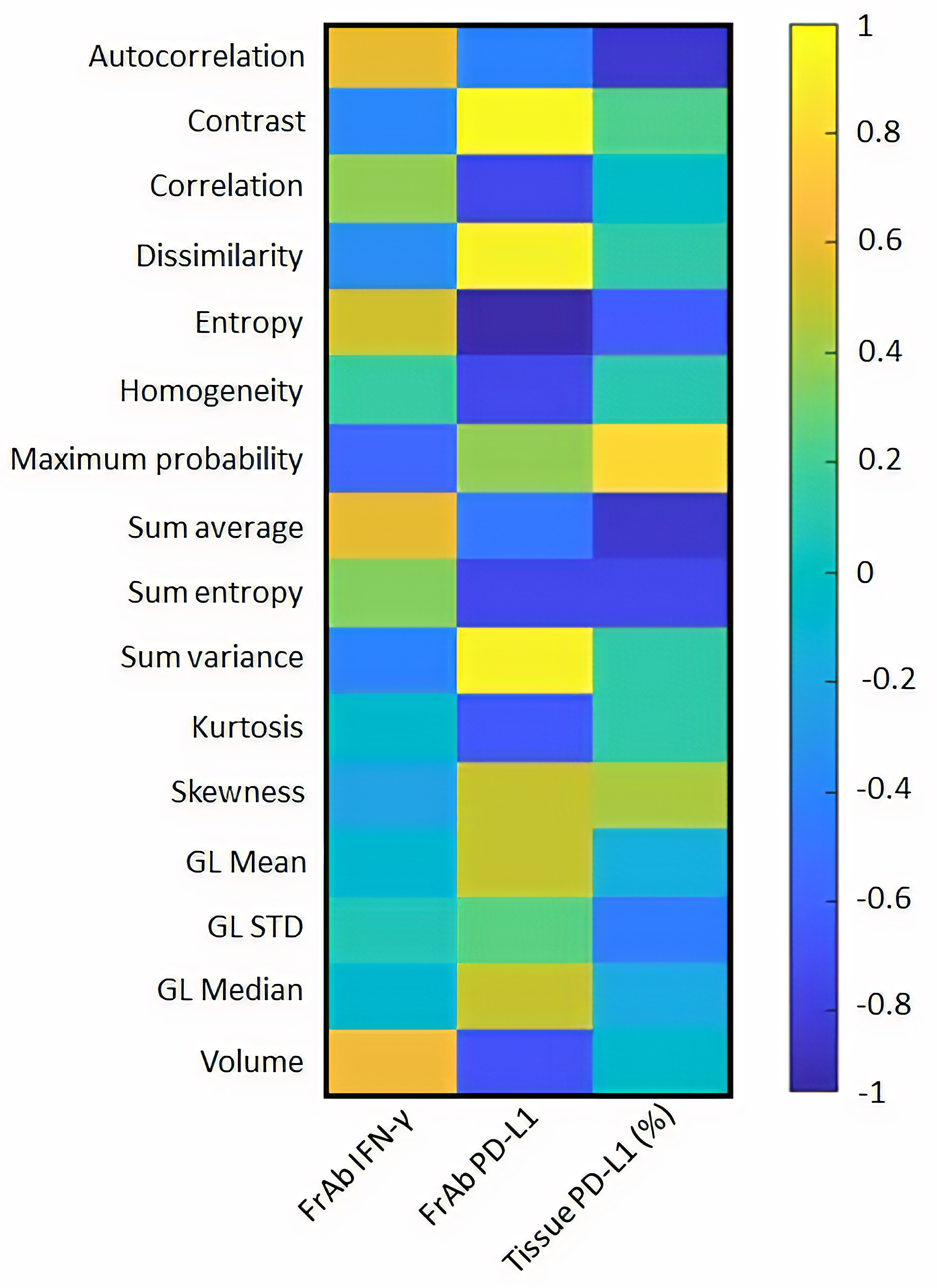

Supplement: Supplementary file 4 — Supplementary file4 (PNG 3657 KB) Supplementary Fig. S1. Heatmaps describing Spearman's correlation between radiomic features and molecular parameters. Bright yellow indicates direct correlation while blue the inverse one, with a gradient from yellow to blue indicating the Spearman's correlation coefficient. Abbreviation: FA, fractional abundance [file 262_2020_2810_MOESM4_ESM.png]
